# Efficacy of an Oral Chondroprotective Joint Supplement on Stride Length and Gait Symmetry in Aged Geldings with Chronic Lameness

**DOI:** 10.3390/ani16081230

**Published:** 2026-04-17

**Authors:** Renee M. Harbowy, Cara I. Robison, Isabella Tillman, Jane M. Manfredi, Brian D. Nielsen

**Affiliations:** 1 Department of Animal Science, Michigan State University, 474 S. Shaw Lane, East Lansing, MI 48824, USA; oconn107@msu.edu (C.I.R.); bdn@msu.edu (B.D.N.); 2 McPhail Equine Performance Center, College of Veterinary Medicine, Michigan State University, East Lansing, MI 48824, USA; tillmani@msu.edu (I.T.); manfred1@msu.edu (J.M.M.)

**Keywords:** glucosamine, chondroitin sulfate, joint health, osteoarthritis, oral joint supplement, equine, avocado/soybean unsaponifiables, soundness

## Abstract

Oral joint supplements (OJSs), specifically those containing glucosamine and chondroitin sulfate, are some of the most popular feed additives fed by horse owners. However, evidence of the efficacy of these nutraceuticals in vivo is lacking. To investigate the impacts of an OJS on equine lameness, 40 geldings (18 ± 7 yr) with chronic lameness and AAEP lameness grades between 2 and 4 (as scored by a veterinary sports medicine and rehabilitation specialist) were enrolled in the study. Horses were stratified by lameness grade, BCS, and weight, and then randomly assigned to receive a placebo powder or an OJS daily for 6 wks. Stride length measurements and lameness evaluations were performed every 2 wk. There was no effect of treatment or treatment-by-day interaction for stride length, though day affected both walk (*p* = 0.04) and trot (*p* < 0.01). Only day affected lameness grade, with the lowest average score on d28 (*p* = 0.04). There was no evidence of supplementation improving gait symmetry over time. As such, there is no evidence to support efficacy of the tested OJS when fed for 6 wk. These results emphasize the importance of well-controlled studies and support continued development of evidence-based management strategies for equine joint health.

## Simple Summary

Equine joint supplements are popular feed additives, mainly due to the prevalence of degenerative joint diseases such as osteoarthritis. Despite their popularity, there is little evidence that these products are efficacious in horses. To evaluate whether a commercially available oral joint supplement could improve joint health, 40 aged geldings with chronic lameness were fed the supplement daily for six weeks. Supplementation did not influence stride length, lameness grade, or objective gait symmetry data. However, changes in these criteria were found over time regardless of treatment. The results of this study indicate that this product is unlikely to improve soundness in lame horses when fed according to the manufacturer’s instructions. These results emphasize the importance of accounting for confounding factors that may influence the presentation of lameness over time and promote the use of evidence-based management strategies for managing equine joint health, such as appropriate diet, housing, and exercise.

## 1. Introduction

Equine osteoarthritis is the leading cause of lameness in horses [1] and is one of the most common chronic diseases in horses [2]. Osteoarthritis is a degenerative joint disease, affecting an estimated 50% of horses over the age of 15 and up to 90% of horses aged 30 or older [3]. Disease progression results in lameness and reduced mobility, subsequently causing compromised performance ability and challenges in maintaining horse comfort [1]. These impacts not only challenge owners financially through treatment cost and lost income but also compromise equine welfare and invite scrutiny to the industry’s social license to operate.

With osteoarthritis being characterized by the degeneration of articular cartilage and structural alteration of the subchondral bone [4], research in recent decades has focused on purported chondroprotective compounds that may be able to counteract damage to the articular cartilage. Namely, glucosamine, chondroitin sulfate, methylsulfonylmethane (MSM), and avocado/soybean unsaponifiables (ASU) have all been shown to produce favorable effects on chondrocytes and subchondral osteoblasts in vitro [5,6,7,8,9,10]. As such, these compounds are amongst the most common active ingredients in equine oral joint supplements (OJSs).

Despite evidence from in vitro trials, the efficacy of these chondroprotective compounds in various combinations in vivo has unclear clinical implications. While some equine studies have reported mild improvements in range of motion following supplementation [11,12], the clinical relevance of these findings is difficult to elucidate. Similarly, in studies evaluating horses with induced osteoarthritis, while benefits were reported for factors such as ultrasonographic joint evaluations and gross joint examinations [13,14], no benefit of treatment was detected in clinical parameters such as range of motion or lameness grade. Conversely, other studies have reported notable improvements in lameness grades, flexion tests, and joint kinematics in response to supplementation [15,16], but these results must be heeded with caution due to the lack of a control group and unbalanced treatment group assignment. As highlighted by these studies, experimental results are often conflicting, or interpretation is limited by the design. Despite decades of research, there remains no conclusive evidence of glucosamine and chondroitin sulfate-based OJS efficacy in horses to date.

The lack of agreement between studies is also noticed by horse owners, with only 48% reporting that they felt the efficacy of supplements is well-researched, despite 90% stating they would feed a joint supplement to treat or prevent joint issues in their horses [17]. Combined with the fact that OJSs are estimated to account for 34% of all horse supplement sales [18], it is critical that owners are fully aware of the impacts—or lack thereof—that these products may have on their animals, both for the financial sake of the owner as well as the welfare of the horse. As such, the objective of this study was to determine the impact of a commercially available OJS on lameness and stride characteristics in aged, exercised geldings with chronic lameness when fed in an on-farm scenario.

## 2. Materials and Methods

### 2.1. Horses, Housing, and Workload

Animal use and the study protocol were approved by the Michigan State University Institutional Animal Care and Use Committee (PROTO202300121). Approximately 60 geldings from a Michigan equestrian summer camp (Black River Farm and Ranch, Croswell, MI, USA) were scored subjectively for lameness using the Association of Equine Practitioners (AAEP) lameness scale [19] by a board-certified equine veterinarian with specialization in surgery, sports medicine, and rehabilitation (ACVS-LA/ACVSMR (Equine)). For initial assessment, horses were trotted on a straight line on an asphalt aisleway (26 m). Of the geldings scored, 40 with AAEP lameness, grades between 2 and 4, with chronic lameness due to previously diagnosed or suspected osteoarthritis, were enrolled into the study. Determination of osteoarthritis was based on horses either having osteoarthritis disclosed when the ranch purchased them or those that were diagnosed by the ranch’s veterinarian. However, confirmatory imaging was not performed as part of this study prior to horse enrollment. Horses were not included if chronic lameness was due to a known or suspected underlying condition other than osteoarthritis.

Selected horses had an average known age of 18 ± 7 yr, body condition score (BCS) of 5 ± 1, and a weight of 494 ± 82 kg. Horses were stratified by these factors, as well as anticipated summer workload (as this study spanned from June to July), to ensure both groups averaged a similar number of working hours per week and were then randomly assigned to either the control (*n* = 20) or OJS treatment (*n* = 20) (Table 1). For the duration of the study, horses were housed on pasture with ad libitum access to pasture, grass hay, and water, apart from when being used for riding and horsemanship classes. Daily horse management and dietary oversight—beyond the items described in Section 2.2—was performed by Black River Farm and Ranch staff under the direction of the ranch’s equine manager. All horses were performing low-intensity work that consisted of walking, trotting, limited cantering, and trail riding.

### 2.2. Control and OJS Powders

The OJS used for this study was a commercial multi-ingredient product containing 14.4 g of glucosamine hydrochloride, 10.0 g of methylsulfonylmethane (MSM), 2.4 g of sodium chondroitin sulfate, 2.1 g of avocado/soybean unsaponifiables (ASU), 0.56 g of *Boswellia serrata* extract, and 0.1 g of manganese, as purported active ingredients per every 32.4 g serving (Cosequin ASU, Nutramax Laboratories, Lancaster, SC, USA). The control powder fed to horses was bleached all-purpose flour, which offered a similar density and consistency to the OJS. The control and OJS groups were further divided into two sub-groups and were assigned different colors to keep the treatments blinded (Table 2). Having four groups allowed for the illusion of four treatments, rather than just two, helping to eliminate any potential bias of handlers and farm staff, as has been previously described [20]. To create the four groups, colored and scented additives were made from cornstarch, water, food-grade dye, and food-grade scented oil. After mixing, the additives were dried and ground back into a powder. The additive was then added into its corresponding bulk control or supplement supply until the desired color and scent-masking was achieved but did not exceed a maximum of 2% of the total supply weight (≤0.3 g/serving) to ensure that the effect on the original product concentration was negligible.

Control or OJS powders were added to a ration balancer (ProElite Grass Advantage, Cargill, Inc., Wayzata, MN, USA; Table 2) and fed for six weeks according to both OJS and ration balancer product directions. The ration balancer was fed according to maintenance recommendations of 0.45 kg/454 kg BW and was composed of 30% crude protein, 6% crude fiber, and 5% crude fat with added micronutrients. The addition of the ration balancer supported horses in meeting nutrient requirements, given that they were primarily maintained on pasture, as well as offering a palatable carrier for the OJS or control powders. This feeding regimen included a 4 wk loading dose of the supplement followed by a 2 wk maintenance dose based on the starting body weight. A loading dose was defined as 32.4 g for horses weighing between 272 and 544 kg (equivalent to two scoops daily), and 48.6 g daily for horses over 544 kg (equivalent to three scoops daily). Maintenance doses were 16.2 g (equivalent to one scoop daily) for horses weighing between 272 and 544 kg, and 32.4 g (equivalent to two scoops daily) for horses over 544 kg. Both loading and maintenance doses were defined by the product manufacturer. There were no observed effects of the scented additive on palatability for any treatment, and all horses were confirmed to have consumed their provision after each feeding.

### 2.3. Sample and Data Collection

Horses were evaluated for their weight, BCS, lameness grade, gait symmetry, and stride length. Sampling and data recording were performed every 2 weeks of the 6 wk supplementation period for a total of four collection timepoints.

### 2.4. Weight and Body Condition Score

All horses were weighed on an electronic scale (Equi3 Weighbridge, Allweigh Scales, Daventry, UK) on flat, compacted dirt flooring on weeks 0, 2, 4, and 6 of supplementation. If a horse would not stand on the scale for an accurate reading, a weight tape was used to get an estimate of the horse’s weight. Horses were also body-condition scored using the Henneke system [21] by the same evaluator every two weeks for the duration of the study.

### 2.5. Subjective Lameness Assessment

Horses were led by the same handler for each evaluation to ensure consistency between runs, and all assessments were completed by the same board-certified veterinarian who performed the initial lameness examinations. An AAEP lameness grade (0 = sound, 5 = non-weight-bearing lameness) was assigned for each limb after watching the horses trot on a straight line on an asphalt surface. Subjective lameness grades were assigned without knowledge of the objective gait analysis findings.

### 2.6. Objective Gait Analysis

Gait symmetry was evaluated concurrently with the subjective lameness examination by using an inertial sensor-based system (Equinosis Q with Lameness Locator, LLC, St. Louis, MO, USA). As previously described and validated for equine gait analysis, the three inertial sensors were placed on the horse’s poll, between the left and right tuber sacrale (pelvis), and the front right pastern to assess the forelimb vector sum (FVS) and the difference in minimum and maximum pelvic height (referred to as PD_min_ and PD_max_, respectively) [22]. The threshold for a front-limb lameness was defined as an FVS ≥ 8.5 mm (refs. [23,24]), with hindlimb lameness defined as a PD_min_ or PD_max_ > 3 mm [22,23].

### 2.7. Stride Length

Stride length was measured at both the walk and trot. For each gait, horses were led in-hand by the same handler over a freshly raked section of a dirt arena. Horses performed three passes at each gait, with the total length of three strides measured for each pass from toe-to-toe impressions [25]. Stride length was then calculated as the average length of a single stride across all three passes. Speed was not controlled in this study to allow assessment of the horses’ preferred natural movement; horses were permitted to set their own pace so long as the correct gait was performed.

### 2.8. Statistical Analysis

A sample size calculation (G*Power 3.1.9.2, Kiel, Germany) was performed based on improvements in forelimb lameness by using an alpha of 0.05, power of 80%, and an expected difference in lameness means of 10 and a standard deviation of 5. The results indicated 36 horses would be required, with additional horses enrolled in case of dropout during the study period.

All other statistical analyses were performed in SAS 9.4 (SAS Institute Inc., Cary, NC, USA). Weight and BCS were analyzed using Proc Mixed with the fixed effects of day, treatment, and treatment by day, with the random effect of horse.

Lameness grades for each limb were analyzed using Proc Glimmix with the fixed effects of treatment, day, and treatment by day, with the random effect of horse. Scores were also evaluated as a composite score, referred to in this study as the total lameness grade, which was calculated as the sum of the scores of all four limbs [25] by using the same statistical model in Proc Mixed.

Stride length was analyzed using Proc Mixed with the fixed effects of treatment, day, treatment by day, and the random effect of horse. Day 0 was included as a significant covariate in the model.

The FVS, PD_min_, and PD_max_ values were analyzed as the absolute value of lameness locator readings to capture the degree of asymmetry without the influence of right- or left-limb lameness origin. Forelimb and hindlimb asymmetries were evaluated using Proc Mixed with the fixed effects of treatment, day, treatment by day, and the random effect of horse. Day 0 measurements were included as a significant covariate for each criterion. Data were also analyzed, including only horses that met the objective lameness criteria at a minimum of one timepoint for the FVS, PD_min_, and PD_max_ values; however, this analysis did not alter the interpretation of the results, so all values were retained.

The results are reported as the least squares mean ± the standard error of the mean (SEM). Significance was set as *p* ≤ 0.05, with tendencies discussed at *p* ≤ 0.1.

## 3. Results

### 3.1. Horse Weight and Body Condition Score

There was no effect of treatment or the interaction of treatment by day for horse weight, but there was a significant effect of day (*p* = 0.003), with horses, on average, weighing less on d42 than on other days, regardless of treatment. There was no effect of treatment, day, or the interaction of treatment by day for BCS.

### 3.2. Subjective Lameness Assessment

Overall, 39 out of the 40 enrolled horses had concurrent forelimb and hindlimb lameness on at least one study date, and one horse had unilateral hindlimb lameness on all study dates. There was no study timepoint in which any horse appeared sound. Scores for the left forelimb, right forelimb, left hindlimb, and right hindlimb had no significant effect of treatment, day, or treatment-by-day interaction. Evaluation as a total lameness score revealed a day effect (*p* = 0.035), with total scores on d28 being lower than those on d0 (*p* = 0.045) and d 14 (*p* = 0.008), but not on d42. There was no effect of treatment or treatment-by-day interaction on the total lameness score (Table 3).

### 3.3. Forelimb Vector Sum (FVS)

The absolute value of the FVS was significantly affected by treatment (*p* = 0.042), with horses in the OJS group having overall greater forelimb asymmetry than those in the control group. There was no effect of day or the interaction of treatment by day (Table 4).

### 3.4. Difference in Minimum Pelvic Height (PD_min_)

There was a treatment-by-day interaction for the absolute value of the difference for minimum pelvic height (*p* = 0.036), with horses in the OJS group tending to have a greater indication of hindlimb asymmetry than the horses in the control group on d14 (*p* = 0.087). There was no effect of treatment or day (Table 5).

### 3.5. Difference in Maximum Pelvic Height (PD_max_)

Neither treatment, day, nor the interaction of treatment by day influenced the absolute value of the difference for maximum pelvic height (*p* = 0.137; *p* = 0.114; and *p* = 0.722, respectively; Table 6).

### 3.6. Stride Length

Treatment and the interaction of treatment by day were insignificant for both the walk and trot, but there was an effect of day for the walk (*p* = 0.048) and trot (*p* < 0.001). Stride length during walking was shorter on d42 than on other days, and stride length during trotting was shorter on d28 than on other days (Table 7).

## 4. Discussion

Despite the popularity of OJSs to treat or prevent joint problems, their efficacy remains unsubstantiated in horses [26,27]. Supplementation did not affect subjective lameness assessments, indicating supplementation of this OJS was not efficacious in ameliorating lameness. This is consistent with findings in an induced osteoarthritis model following ASU supplementation [13]. In contrast, other controlled studies supplementing glucosamine and chondroitin sulfate have found differences in lameness grade between treatments, though only on d120 of the study [14]. It is important to note that subjective lameness exams using the AAEP scale may be influenced by the rater, and inter-rater agreements often vary [28,29]. It is this subjectivity that may contribute to conflicting results between studies. However, this assessment criterion continues to be an important study inclusion due to its continued use by clinically practicing veterinarians and ease of understanding for horse owners.

This caveat of subjective lameness exams has promoted the use of more objective lameness assessment methods, fulfilled in this study with an inertial sensor system. Objective assessment of gait symmetry aligned with subjective lameness exam results, indicating no improvement in lameness in response to supplementation for the FVS, PD_min_, or PD_max_ values. In fact, though an overall treatment effect was found for the FVS values, it appeared that horses in the OJS treatment group had a greater indication of forelimb asymmetry than control horses, irrespective of the study day. Similarly, the treatment-by-day interaction for the PD_min_ values also indicated that horses receiving the OJS had a greater indication of hindlimb asymmetry than the control horses on d14. While these findings may be a statistical artifact, as horses were stratified by subjective lameness scores and not an objective measurement of asymmetry, the lack of a treatment-by-day interaction for the FVS values indicated no change in the forelimb asymmetry of treated horses over time. Further, no day differences between treatments were found on d28 or d42 of supplementation for the PD_min_ values, nor was there a difference in d28 or d42 within the OJS-treated horses. Additionally, there was no treatment or day effects on the PD_max_ values. As such, the provision of a joint supplement did not improve the objective measures of gait symmetry, which aligns with previous findings of glucosamine-chondroitin sulfate supplementation [14]. While some previous studies have found moderate impacts of oral joint supplements on joint kinematics [12,16], others have found no influence of supplementation [30], though the overall evaluation of gait symmetry was not evaluated in these latter studies. Based on the results of the present study, it is unlikely that the purported active ingredients commonly included in these supplements elicit detectable alterations in gait.

There was no effect of treatment, day, or the interaction thereof on lameness grade for each individual limb. When evaluated as a total lameness score, there was a significant effect of day, but there was no influence of treatment or the interaction of treatment by day. Similarly, stride length was found to be influenced by day but not by treatment, nor by the interaction of treatment by day. While an analysis of the stride rate could have been beneficial, an assessment of the stride length at a self-set speed within a gait reflects how the horses were most comfortable moving. This methodology is valuable for interpreting the effects of supplementation on lameness-related changes in gait and has been used in previous studies [31,32]. As such, supplementation did not influence the lameness grade or stride length. These results emphasize the importance of accounting for time in studies evaluating lameness in horses. Not only may variations in total lameness scores and stride length be due to progression of the summer season (June through July) and therefore the amount of time the horses had spent in their summer work program, but the degree of lameness is known to fluctuate over time due to a multitude of factors [33,34]. Particularly, though osteoarthritis is known to be a progressive, degenerative disease, symptom presentation is also known to vary across time [2,35]. The findings of the present study align with those previously mentioned and should encourage horse owners to carefully consider time-related or environment-related changes in lameness that may influence their perception of supplement efficacy.

Though the horses did have a lower average weight on d42 regardless of treatment, there was no effect of treatment or time on BCS. As such, it is suspected that the change in horse weight is likely due to water loss, dually attributed to the reduced moisture content of pasture as the summer progressed, as well as horses likely sweating more around the 6-week time point (around mid-July in MI, USA), given that this detected weight change was not apparent in the horses’ body condition. Further, the weight loss on d42 was not associated with improvement in any parameter. However, though this did not influence the results of this study, this once again emphasizes the importance of being cognizant of external factors that have the potential to influence one’s perception of supplement efficacy.

Though this study did not include an assessment of serum or synovial fluid biomarkers related to cartilage metabolism, previous in vivo studies have not been able to definitively detect alterations in anabolic or catabolic activity of articular cartilage post-supplementation [12,13,14,36,37]. While these biomarkers would be advantageous to further characterize the potential mechanisms of purported chondroprotective compounds in vivo, the downstream physiological outcomes on equine performance in response to supplementation remain of greatest value to the horse owner. Further, the ideal dosage and feeding duration of these compounds remains to be determined in the horse. As such, justification of tested concentrations and feeding durations is a common limitation in evaluating oral joint supplements in horses [26], including the present study. While it is possible that 6 weeks may not have been a sufficient duration to detect a change in this group, the chosen study duration was based on the manufacturer’s implication that a change can be detected within 4 weeks. It is also important to note that horses enrolled in this study did vary in terms of lameness grade and the number of limbs affected, rather than a homogenous lameness presentation. While an assessment of specific joints or an evaluation of horses with a consistent presentation of lameness might aid in elucidating a product’s effect, it is likely that many horses fed these products have multi-joint involvement of OA. Overall, though the authors acknowledge the limitations of this study, which can be built upon in future work, the use of a commercially available product in accordance with the manufacturer’s recommendation in horses with varying lameness presentations allows for a much-needed assessment of the efficacy of this OJS in an applied, on-farm scenario.

Based on the results of the present study, 6 weeks of supplementing a commercially available OJS was not effective in reducing signs of lameness in treated horses compared to their controlled counterparts. As such, it is unlikely that oral joint supplements of this composition can benefit horses that have already developed chronic lameness because of degenerative joint diseases, such as osteoarthritis, within 6 weeks. Continued research may be warranted to establish appropriate dosing regimens or evaluate the impact of prophylactic use of purported chondroprotective compounds on equine joint health. However, current management of equine joint health and maintenance of osteoarthritic horses should be based on evidence-based strategies that are focused on appropriate diet and exercise regimens.

## 5. Conclusions

Oral joint supplements are noted as a major component of equine supplement sales, and a vast majority of horse owners have reported using joint supplements to treat or manage their horses’ joint health. This is largely attributed to the negative impacts that equine degenerative joint diseases, namely osteoarthritis, have on equine performance and welfare. While equine chondroprotective compounds show some promise in vitro, studies evaluating the efficacy of these compounds in the horse are largely limited. Based on the results of the present study, it is unlikely that this equine oral joint supplement, composed of glucosamine hydrochloride, MSM, sodium chondroitin sulfate, ASU, *Boswellia serrata* extract, and manganese, is efficacious in improving lameness in aged geldings following 6 weeks of supplementation. While some parameters showed changes over time, no beneficial treatment effects were detected for subjective lameness grade, gait symmetry, or stride length. While limitations remain in the evaluation of equine oral joint supplements, particularly with dosage and ideal feeding durations, it is important for horse owners to be wary of the confounding factors that may influence a horse’s lameness presentation over time. Best practices for managing equine joint health remain with evidence-based strategies such as appropriate dietary design, housing, and exercise rather than the use of purported chondroprotective nutraceuticals.

## Figures and Tables

**Table 1:** Average ± standard error of the mean (SEM) of lameness grade, known age (yr), weight (kg), and body condition score (BCS) of horses within each treatment group.

Treatment	Lameness Grade	Age (yr)	Weight (kg)	BCS
Control	2.5 ± 0.1	16.8 ± 1.3	497 ± 16	5 ± 0.2
OJS	2.5 ± 0.1	19.5 ± 2.0	490 ± 21	5 ± 0.2

**Table 2:** Color and scent additives for oral joint supplement (OJS) and control treatment groups.

Treatment	Additive Color	Additive Scent	Number of Horses
OJS	Red	Peppermint	10
	None	None	10
Control	Green	Green Apple	10
	Orange	Orange	10

**Table 3:** Mean total lameness grade (sum of all AAEP assigned lameness scores per limb) by treatment and day (*n* = 20 per treatment).

	Day				
Treatment	0 ^a^	14 ^a^	28 ^b^	42 ^ab^	SEM
Oral Joint Supplement (OJS)	4.9	4.9	4.4	4.7	0.2
Control	4.7	5.0	3.9	4.1	0.2
SEM	0.2	0.2	0.2	0.2	-

^a,b^ Days lacking common superscripts differ at *p* ≤ 0.05. SEM = standard error of the mean.

**Table 4:** Absolute value of the mean front vector sum (FVS) in mm by treatment and day (*n* = 20 per treatment).

	Day			
Treatment	14	28	42	SEM
Oral Joint Supplement (OJS) *	22.2	18.6	10.1	3.0
Control	13.4	13.7	17.1	3.0
SEM	2.1	2.1	2.1	-

* Indicates that overall treatment means differ at *p* ≤ 0.05. Day 0 values were included as a significant covariate in the model. SEM = standard error of the mean.

**Table 5:** Absolute value of difference in mean minimum pelvic height (PD_min_) in mm by treatment and day (*n* = 20 per treatment).

	Day			
Treatment	14	28	42	SEM
Oral Joint Supplement (OJS)	7.6 †	5.5	6.0	1.2
Control	4.8	5.5	5.3	1.2
SEM	0.8	0.8	0.8	-

† Indicates that OJS horses have marginal evidence of differing from control horses within day 14 at *p* ≤ 0.1. Day 0 values were included as a significant covariate in the model. SEM = standard error of the mean.

**Table 6:** Absolute value of difference in mean maximum pelvic height (PD_max_) in mm by treatment and day (*n* = 20 per treatment).

	Day			
Treatment	14	28	42	SEM
Oral Joint Supplement (OJS)	10.1	7.9	7.6	1.6
Control	6.2	5.4	4.8	1.6
SEM	1.1	1.1	1.1	-

Day 0 values were included as a significant covariate in the model. SEM = standard error of the mean.

**Table 7:** Mean stride length (m) at both the walk and trot by treatment and day (*n* = 20 per treatment).

	WALK			
	Day			
Treatment	14 ^a^	28 ^a^	42 ^b^	SEM
OJS	1.53	1.54	1.53	0.01
Control	1.58	1.57	1.55	0.01
SEM	0.01	0.01	0.01	-
	**TROT**			
	**Day**			
**Treatment**	**14 ^a^**	**28 ^b^**	**42 ^a^**	**SEM**
OJS	1.92	1.83	1.89	0.02
Control	1.97	1.88	1.92	0.02
SEM	0.02	0.02	0.02	-

^a,b^ Days lacking common superscripts within each gait differ at *p* ≤ 0.05.

## Data Availability

The original contributions presented in this study are included in the article/Supplementary Material. Further inquiries can be directed to the corresponding author.

## Supplementary Materials

The following supporting information can be downloaded at: https://www.mdpi.com/article/10.3390/ani16081230/s1.

## Abbreviations

The following abbreviations are used in this manuscript:

ASU	Avocado/soybean unsaponifiables
BCS	Body condition score
FVS	Forelimb vector sum
PD_max_	Difference in maximum pelvic height
PD_min_	Difference in minimum pelvic height
MSM	Methylsulfonylmethane
OJS	Oral joint supplement
